# Machine learning models for predicting hepatocellular carcinoma development in patients with chronic viral hepatitis B infection

**DOI:** 10.2478/abm-2025-0007

**Published:** 2025-02-28

**Authors:** Warissara Kuaaroon, Thodsawit Tiyarattanachai, Terapap Apiparakoon, Sanparith Marukatat, Natthaporn Tanpowpong, Sombat Treeprasertsuk, Rungsun Rerknimitr, Pisit Tangkijvanich, Prooksa Ananchuensook, Watcharasak Chotiyaputta, Kittichai Samaithongcharoen, Roongruedee Chaiteerakij

**Affiliations:** Faculty of Medicine, Chulalongkorn University, Bangkok 10330, Thailand; Department of Computer Engineering, Faculty of Engineering, Chulalongkorn University, Bangkok 10330, Thailand; Image Processing and Understanding Team, Artificial Intelligence Research Group, National Electronics and Computer Technology Center, Pathumthani 12120, Thailand; Department of Radiology, Faculty of Medicine, Chulalongkorn University and King Chulalongkorn Memorial Hospital, Thai Red Cross Society, Bangkok 10330, Thailand; Division of Gastroenterology, Department of Medicine, Faculty of Medicine, Chulalongkorn University and King Chulalongkorn Memorial Hospital, Thai Red Cross Society, Bangkok 10330, Thailand; Center of Excellence for Innovation and Endoscopy in Gastrointestinal Oncology, Chulalongkorn University, Bangkok 10330, Thailand; Center of Excellence in Hepatitis and Liver Cancer, Department of Biochemistry, Faculty of Medicine, Chulalongkorn University, Bangkok 10330, Thailand; Division of Gastroenterology, Department of Medicine, Faculty of Medicine Siriraj Hospital, Mahidol University, Bangkok 10700, Thailand

**Keywords:** artificial intelligence, chronic viral hepatitis B infection, clinical prediction model, Extreme Gradient Boosted model, HCC surveillance

## Abstract

**Background:**

Chronic hepatitis B (CHB) infection is the major risk factor for hepatocellular carcinoma (HCC).

**Objective:**

To develop machine-learning models for predicting an individual risk of HCC development in CHB-infected patients.

**Methods:**

Machine learning models were constructed using features from follow-up visits of CHB patients to predict the diagnosis of HCC development within 6 months after each index follow-up. We developed 4 model variants using all features, with alpha fetoprotein (AFP) (*AF ^A^*) and without AFP (*AF^N^*); and selected features, with AFP (*SF ^A^*) and without AFP (*SF^N^*). Performance was evaluated using 10-fold cross-validation on a derivation cohort and further validated on an independent cohort.

**Results:**

In the derivation cohort of 2,382 patients, of whom 117 developed HCC, *AF^A^* achieved higher sensitivity (0.634, 95% confidence interval [CI]: 0.559–0.708) and specificity (0.836; 0.830–0.842) than *AF ^N^* (sensitivity 0.553; 0.476–0.630 and specificity 0.786; 0.779–0.792). *SF^A^* also achieved higher sensitivity (0.683; 0.611–0.755 vs. 0.658; 0.585–0.732) and specificity (0.756; 0.749–0.763 vs. 0.744; 0.737–0.751) than *SF^N^*. Performance of *SF^A^* and *SF^N^* were tested in another cohort of 162 patients in which 57 patients developed HCC. *SF^A^* achieved sensitivity and specificity of 0.634 (0.522–0.746) and 0.657 (0.615–0.699), while sensitivity and specificity of *SF^N^* were 0.690 (0.583–0.798) and 0.651 (0.609–0.693), respectively.

**Conclusion:**

The machine learning models demonstrate good performance for predicting short-term risk for HCC development and may potentially be used for tailoring surveillance interval for CHB patients.

Chronic viral hepatitis B (CHB) infection is a major health problem worldwide, with 257 million reported cases in 2015 [1]. Patients with CHB infection have a significant risk of developing hepatocellular carcinoma (HCC), that is, approximately 53% of HCC cases were globally attributed to CHB [2]. In East Asia, the HCC incidence rate among inactive viral hepatitis B carriers was 0.2 per 100 person-years, with a 5-year cumulative incidence of 1%. The incidence rate increased to 0.6 and 3.7 per 100 person-years, with 5-year cumulative incidences of 3% and 17% in CHB-infected patients who had chronic active hepatitis without cirrhosis and those with compensated cirrhosis, respectively [3].

International guidelines recommend that CHB-infected patients should be surveilled for HCC by ultrasound with or without serum tumor biomarker alpha fetoprotein (AFP) test every 6 months [4,5,6]. This recommendation is based on the tumor volume doubling time of approximately 4–5 months. Nonetheless, the risk of HCC development substantially varied among individuals. Therefore, the screening interval of 6 months may not be optimal for all CHB-infected patients and should be tailored based on the magnitude of HCC risk of each individual patient. Various risk scores were proposed to stratify the risk of HCC development in CHB-infected patients. The Guide with age, gender, hepatitis B virus (HBV) DNA, Core Promoter Mutations and Cirrhosis-HCC (GAG HCC), and Chinese University-HCC (CU-HCC) were developed to predict 5- and 10-year risk, while Liver Stiffness Measurement-HCC (LSM-HCC) and Risk Estimation for Hepatocellular Carcinoma in Chronic Hepatitis B (REACH-B) were developed using traditional regression analysis for predicting 3- and 5-year risk [7,8,9,10]. Recently, Plan-B (Prediction of Liver cancer using Artificial intelligence-driven model for Network-hepatitis B) and the deep-learning-based model were developed as machine learning models for predicting HCC in CHB patients [11, 12]. However, these scores predict long-term risk and therefore have a minimal impact on deciding upon a shorter-term HCC surveillance interval.

This study aimed to develop machine learning models to predict a short-term risk for development of HCC, that is, within 6 months after each follow-up visit. The secondary aim was to validate performance of the developed models in another patient cohort. These models could potentially customize the surveillance interval for individuals with CHB infection.

## Methods

This retrospective study was approved by the Institutional Review Board of Chulalongkorn University (IRB No. 0263/2022), and the requirement for informed consent was waived due to the retrospective nature of the study. The study was performed in accordance with the Declaration of Helsinki. All patient data were analyzed anonymously.

### Dataset

We collected two datasets, one for derivation of the machine learning models and the other for validation of the model performance, as follows:

### Derivation dataset

Derivation dataset was used for developing the model and internally evaluating the model performance. We retrieved patient demographics, International Classification of Diseases (ICD)-10 diagnosis codes, and laboratory data and radiology reports through the Hospital Information System (HIS) and Radiology Information System (RIS) of the King Chulalongkorn Memorial Hospital, Bangkok, Thailand between January 2008 and December 2020.

Patients with CHB were identified using the ICD codes B18.0, B18.1, B18.8, or B18.9 or a positive test for hepatitis B surface antigen (HBsAg). HCC diagnosis was defined by ICD codes C22.0, C22.7, or C22.9. We also manually reviewed patients' electronic medical records to verify the diagnosis of HCC as well as the dates of HBV infection and HCC diagnosis. Patients diagnosed with HCC within 6 months after the date of HBV diagnosis were excluded because it was possible that HCC existed prior to the date of the patient being diagnosed with HBV infection. The presence of cirrhosis was retrieved from upper abdominal ultrasound reports or ICD codes K74.0, K74.6 (**Table 1**). Other retrieved laboratory data were hepatitis C virus (HCV) and HIV infection, hepatitis B and C viral loads, hepatitis B e antigen (HBeAg) and antibody (anti-HBe), AFP level, liver and renal function tests, blood glucose, complete blood counts, and coagulation studies. Data from all follow-up visits starting from the date of HBV infection diagnosis to the date of HCC diagnosis or the end of study period (December 2020) were used for constructing the predictive models. The primary outcome was development of HCC within the next 6 months from each visit.

**Table 1.** Patient demographic, laboratory, and radiologic parameters used as inputs to the models

**Parameters**	**Pearson's correlation coefficient**
Age^*^	0.047
Sex^*^	0.025
HBV viral load	0.007
HbeAg	0.018
Anti-HBe	0.004
Anti-HCV	0.024
HCV-RNA (Positive/Negative)	0.030
Anti-HIV	0.004
AFP (for *AF^A^* and *SF^A^*)	0.180
TB^*^	0.095
DB	0.096
AST	0.052
ALT	0.011
ALP^*^	0.120
Albumin	0.029
Globulin	0.071
INR^*^	0.130
Hemoglobin	0.037
Total white blood cell count	0.024
Absolute neutrophil count	0.058
Absolute lymphocyte count	0.017
Platelet count	0.006
BUN	0.060
Cr	0.033
FPG	0.017
Hemoglobin A1C	0.020
Liver cirrhosis (present/absent)^*^	0.075
Liver steatosis (present/absent)^*^	0.040

* These features were included as a set of selected features.

AFP, alpha fetoprotein; ALP, alkaline phosphatase; ALT, alanine aminotransferase; AST, aspartate aminotransferase; BCLC, Barcelona Clinic Liver Cancer; BUN, blood urea nitrogen; Cr, creatinine; DB, direct bilirubin; FPG, fasting plasma glucose; HBV, hepatitis B virus; HBeAg, hepatitis B e antigen; HCV, hepatitis C virus; INR, international normalized ratio; IQR, interquartile range; SD, standard deviation; TB, total bilirubin.

### External validation dataset

Another dataset obtained from the Faculty of Medicine Siriraj Hospital, Mahidol University, was used for externally validating the performance of the predictive models. This dataset was obtained by a case–control method. Patients with CHB who developed HCC served as cases. For controls, we identified CHB patients who had not developed HCC, then matched with cases in a 1:1 ratio by the time period of visit at the Hepatology clinic. The data were manually reviewed and abstracted from medical records by 2 experienced hepatologists. The same inclusion and exclusion criteria of the derivation dataset were applied to the validation dataset.

### Development of machine learning models for HCC prediction

We trained Extreme Gradient Boosted (XGBoost) models using clinical, laboratory, and radiologic parameters in each follow-up as inputs to predict HCC development within 6 months after each index follow-up.

A total of 28 features were included as “set of all features”, consisting of the following features: sex, age, quantitative HBV viral load, Hepatitis B e Antigen (HBeAg), anti-HBe, anti-HCV, qualitative HCV viral load, anti-HIV, AFP, total bilirubin (TB), direct bilirubin (DB), aspartate transaminase (AST), alanine aminotransferase (ALT), alkaline phosphatase (ALP), albumin, globulin, hemoglobin (Hb), white blood cell count (WBC), absolute neutrophil count, absolute lymphocyte count, platelet count, international normalized ratio (INR), blood urea nitrogen (BUN), creatinine (Cr), fasting plasma glucose (FPG), HbA1C, presence of cirrhosis, and liver steatosis status.

We acknowledged that selecting only some important features as model inputs may enable more practical use of the models by clinicians, as the set of all features may not always be completely available during the follow-up visit, and focusing on only important features can save time and effort of clinicians in inputting data into the models. Thus, we further identified features that had a high correlation with the outcome of interest (i.e., HCC development) determined by Pearson correlation coefficient in the derivation dataset. Among features that exhibited multicollinearity, we kept only a single feature that had the highest correlation with the outcome. As a result, 3 of the most highly correlated features, namely TB (Pearson correlation coefficient 0.095), ALP (0.120), and INR (0.130), along with 4 clinically important *A Priori* features (age [0.047], sex [0.025], liver cirrhosis [0.075], and liver steatosis [0.040]), were included as a “selected feature set” (7 features in total) (**Table 1**).

**Figure 1.**
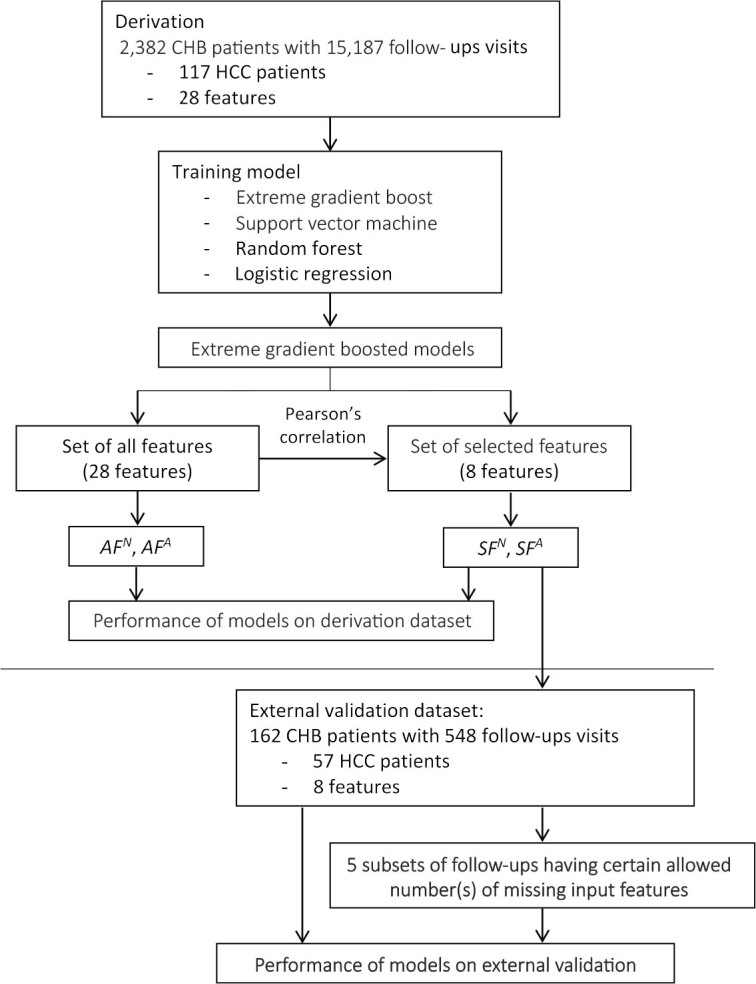
Flow diagram of the study. *AF^A^*, represents the model using a set of all features, with AFP, as inputs. *AF^N^*, represents the model using a set of all features, without AFP, as inputs. *SF^A^*, represents the model using a set of selected features, with AFP, as inputs. *SF^N^*, represents the model using a set of selected features, without AFP, as inputs; HCC: Hepatocellular carcinoma.

Considering that serum AFP is an important feature associated with HCC development [13], the AFP test is not always available, especially in hospitals located in remote areas; we therefore developed two model variants: a model with AFP as an input feature and a model without AFP as an input feature, in order to enable the model to be applied in a situation when the AFP test is not performed. To summarize, the following notations are used in the remaining of this article (**Figure 1**).

### Statistical analysis

All analyses were performed using Python version 3.7.3 (Python Software Foundation, Delaware, Beaverton, Oregon, US). XGBoost library implementation of Gradient Boosted Trees was used to construct the model. Machine learning deeply learned all training data and identified relationships between the data and the diagnosis of HCC, which might not be detected by traditional statistics, then developed conditions and rules for HCC predictive models. Using the derivation dataset, we performed 10-fold cross-validation to estimate the performance of the *AF ^A^*, *AF^N^*, *SF ^A^*, and *SF^N^* models. In each fold, 90% of the dataset were used for training and 10% were used for performance evaluation. Data split in each fold was independent at patient level. Sensitivities, specificities, and area under receiver operating characteristic curve (AUROC) along with 95% confidence intervals (CIs) of the models for predicting HCC development were estimated. For each ROC, a cutoff point that yielded maximum sensitivity was used to obtain the sensitivity and specificity of the model. AUROC on the derivation dataset evaluated by 10-fold cross-validation was reported as mean ± standard deviation (SD) of AUROCs across all 10 folds. Next, the performance of the *AF^A^* and *SF^N^* model was evaluated on the external validation dataset. AUROCs on the external validation dataset was reported along with 95% CIs. The two-tailed statistical significance level (α) was set at 0.05.

Because the data were retrospectively abstracted from medical records, some input features were inevitably missing. To evaluate how the number of missing input features affected the model performance, we further evaluated the model performance using a set of selected features on subsets of follow-ups having certain allowed number(s) of missing input features. For example, in the subset of follow-ups having 2 allowed missing input features, follow-ups having 0, 1, and 2 missing input features were included. Of note, all follow-ups had at least 4 features, that is, sex, age, cirrhosis, and liver steatosis status, as these features were always available in the medical records. Therefore, the maximum numbers of allowed missing features were three for *SF^N^* (INR, ALP, TB) and four for *SF^A^* (AFP, INR, ALP, TB) (**Figure 1**).

## Results

### Baseline characteristics

#### Derivation dataset

The derivation dataset comprised 2,382 patients with 15,187 follow-up visits. There were 1,331 (55.1%) males with a mean age of 51.0 (SD: 14.7) years. Cirrhosis and HCV co-infection were present in 609 (25.6%) and 65 (2.7%) patients, respectively. There were 11,837 follow-ups with AFP values and 3,350 follow-ups without AFP values. During a median follow-up time of 18 months (interquartile range [IQR]: 52), 117 (4.9%) patients developed HCC. Of the 117 HCC patients of whom 91 (77.8%) had information on HCC stage available, 26 (28.6%) patients were diagnosed HCC at Barcelona Clinic Liver Cancer (BCLC) stage 0, and 44 (48.4%), 10 (11.0%) and 11 (12.1%) were diagnosed at BCLC stage A, B, and C, respectively (**Table 2**).

**Table 2.** Baseline characteristics of patients in the derivation dataset and external validation dataset

	**Derivation dataset**	**Validation dataset**
Total patients, n	2,382	162
Total follow-ups visit, n	15,187	564
Median follow-up time (IQR), month	18.0 (52.0)	11.2 (8.7)
Patients developing HCC, n (%)	117 (4.9%)	57 (35.2%)
BCLC stage^*^, n (%)		
stage 0	26 (28.6%)	13 (22.8%)
stage A	44 (48.4%)	32 (56.1%)
stage B	10 (11.0%)	10 (17.5%)
stage C	11 (12.1%)	2 (3.5%)
Age, mean (SD) (years)	51.0 (14.7)	58.0 (13.6)
Male, n (%)	1,331 (55.1%)	104 (64.2%)
Cirrhosis, n (%)	609 (25.6%)	57 (35.2%)
HCV co-infection, n (%)	65 (2.7%)	0 (0.0%)

* 26 HCC patients in the derivation dataset had no information on BCLC stage available.

BCLC, Barcelona Clinic Liver Cancer; HCC, hepatocellular carcinoma; HCV, hepatitis C virus; IQR, interquartile range; SD, standard deviation.

#### External validation dataset

The dataset comprised 162 CHB-infected patients with 564 follow-ups. In this dataset, 104 (64.2%) patients were male and the mean age was 58.0 (SD: 13.6) years. Cirrhosis was present in 57 (35.2%) patients. None had HCV coinfection. There were 355 and 209 follow-ups with and without AFP values, respectively. During a median follow-up time of 11.2 (IQR: 8.7) months, 57 (35.2%) patients developed HCC; and 13 (22.8%), 32 (56.1%), 10 (17.5%), and 2 (3.5%) patients were diagnosed at BCLC stages 0, A, B, and C, respectively (**Table 2**).

### Performance of the machine learning models for HCC prediction

#### Performance on the derivation dataset

Performance of the models on the derivation dataset are demonstrated in **Table 3**. In the models with a set of all features, *AF^A^* achieved a better sensitivity and specificity than *AF^N^*, with sensitivities of 0.634 (95% CI: 0.559–0.708) and 0.553 (95% CI: 0.476–0.630); and specificities of 0.836 (95% CI: 0.830–0.842) and 0.786 (95% CI: 0.779–0.792), for *AF^A^* and *AF^N^*, respectively. A similar trend was detected for the models using a set of selected features as inputs, however, with less magnitude of difference in performance between the models with and without AFP, that is., *SF^A^* and *SF^N^* had a sensitivity of 0.683 (95% CI: 0.611–0.755) and 0.658 (95% CI: 0.585–0.732); and a specificity of 0.756 (95% CI: 0.749–0.763) and 0.744 (95% CI: 0.737–0.751), respectively. Overall, the models with all features achieved lower sensitivities but higher specificities than the models with selected features. Among the four models, *AF^A^* achieved the highest mean ± SD AUROC of 0.786 ± 0.113, followed by *AF^N^* (0.731 ± 0.089), *SF^A^* (0.727 ± 0.097) and *SF^N^* (0.707 ± 0.088) (**Table 3**).

**Table 3.** Performance of machine learning models for HCC prediction in the derivation dataset and the external validation dataset

	**Derivation dataset**			**External validation dataset**		

	**Sensitivity (95% CI)**	**Specificity (95% CI)**	AUROC^*^ mean ± SD	**Sensitivity (95% CI)**	**Specificity (95% CI)**	**AUROC (95% CI)**
**Models with all features** (***AF***)						
With AFP (*AFA*)	0.634 (0.559–0.708)	0.836 (0.830–0.842)	0.786 ± 0.113	NA	NA	NA
Without AFP (*AFN*)	0.553 (0.476–0.630)	0.786 (0.779–0.792)	0.731 ± 0.089	NA	NA	NA
**Models with selected features** (***SF***)						
With AFP (*SFA*)	0.683 (0.611–0.755)	0.756 (0.749–0.763)	0.727 ± 0.097	0.634 (0.522–0.746)	0.657 (0.615–0.699)	0.646 (0.585–0.706)
Without AFP (*SFN*)	0.658 (0.585–0.732)	0.744 (0.737–0.751)	0.707 ± 0.088	0.690 (0.583–0.798)	0.651 (0.609–0.693)	0.663 (0.605–0.721)

* AUROC is reported as mean ± SD of AUROCs across all 10 folds of cross-validation.

AFP, alpha fetoprotein; AUROC, area under receiver operating characteristic curve; 95% CI, 95% confidence interval; HCC: Hepatocellular carcinoma; NA, not applicable; SD, standard deviation.

#### Performance on the external validation dataset

In contrast to the derivation dataset, *AF^A^* achieved a lower sensitivity than *SF^N^*, that is, 0.634 (95% CI: 0.522–0.746) and 0.690 (95% CI: 0.583–0.798), respectively. Specificities of the two models were relatively similar, that is, 0.657 (95% CI: 0.615–0.699) and 0.651 (95% CI: 0.609–0.693), for *AF^A^* and *SF^N^*, respectively. The AUROC of *AF^A^* and *SF^N^* were 0.646 (95% CI: 0.585–0.706) and 0.663 (95% CI: 0.605–0.721), respectively (**Table 3**).

#### Effects of missing features on model performance

We evaluated the *AF^A^* and *SF^N^* models on subsets of follow-ups having varied numbers of missing input features (**Table 4**). Without missing features, *AF^A^* and *SF^N^* achieved sensitivities of 0.833 (95% CI: 0.535–1.000) and 0.889 (95% CI: 0.684–1.000). We found that the sensitivities of both models progressively decreased by the numbers of missing features, that is, *AF^A^* achieved sensitivities of 0.720 (95% CI: 0.544–0.896), 0.698 (95% CI: 0.560–0.835), 0.647 (95% CI: 0.533–0.761), and 0.634 (95% CI: 0.522–0.746) when the maximum numbers of missing features were 1, 2, 3, and 4, respectively. Likewise, *SF^N^* achieved sensitivities of 0.727 (95% CI: 0.575–0.879), 0.708 (95% CI: 0.580–0.837), and 0.690 (95% CI: 0.583–0.798) when the maximum numbers of missing features were 1, 2, and 3, respectively. These observations indicated that the models achieved better sensitivities with lower numbers of missing input features.

**Table 4.** Performance of machine learning models in the external validation dataset when some features were missing

**Maximum number of missing features**	**Model with AFP (*AF^A^*)**			**Model without AFP (*SF^N^*)**		
	
	**Sensitivity (95% CI)**	**Specificity (95% CI)**	**AUROC (95% CI)**	**Sensitivity (95% CI)**	**Specificity (95% CI)**	**AUROC (95% CI)**
0	0.833 (0.535–1.000)	0.476 (0.263–0.690)	0.655 (0.458–0.851)	0.889 (0.684–1.000)	0.522 (0.318–0.726)	0.705 (0.554–0.856)
1	0.720 (0.544–0.896)	0.670 (0.580–0.759)	0.695 (0.594–0.795)	0.727 (0.575–0.879)	0.644 (0.564–0.725)	0.683 (0.595–0.772)
2	0.698 (0.560–0.835)	0.631 (0.559–0.702)	0.664 (0.586–0.742)	0.708 (0.580–0.837)	0.592 (0.524–0.660)	0.639 (0.564–0.713)
3	0.647 (0.533–0.761)	0.602 (0.551–0.652)	0.624 (0.562–0.687)	0.690 (0.583–0.798)	0.651 (0.609–0.693)	0.663 (0.605–0.721)
4	0.634 (0.522–0.746)	0.657 (0.615–0.699)	0.646 (0.585–0.706)	NA	NA	NA

AFP, alpha fetoprotein; AUROC, area under receiver operating characteristic curve; 95% CI, 95% confidence interval; NA, not applicable; SD, standard deviation.

## Discussion

In this study, we developed machine learning models using clinical parameters to predict the short-term risk of developing HCC in CHB-infected patients. We developed models with various configurations in order to facilitate clinical applications in different scenarios.

In the process of predictive model development, we considered clinical information and laboratory data that are usually monitored during follow-up visits in routine practice as model inputs. Crafting input features in machine learning models is an important step toward obtaining a useful model. Although Gradient Boosted Trees inherently select features with high discriminating power at each decision point, selecting some important features as inputs could enhance clinical applicability of the models, because some laboratory values may not always be available at every follow-up visit. Besides, it may be more practical and time- and effort-saving for clinicians to input only some features into the models during their point-of-care services. Our results showed that the models with a set of selected input features (*SF^A^* and *SF^N^*) had comparable performance to those models with a set of all input features (*AF^A^* and *AF^N^*), supporting the use of models with selected input features in practice.

We also investigated effects of missing features on the model performance and found that the performance was better with fewer numbers of missing input features. Considering selected features, including TB, ALP, INR, AFP, age, sex, cirrhosis, and liver steatosis status, we believe that these laboratory and clinical parameters are usually available in the clinic, further supporting the clinical applicability of our models.

AFP was previously shown to improve screening and surveillance of HCC, when added to ultrasound [14]. Accordingly, we developed variants of models with or without AFP as input features. The gain in performance upon adding AFP as a feature in our models was observed. We therefore recommend monitoring AFP values during follow-up in order to fully utilize the best-performing models. Models without AFP also produced acceptable sensitivities and specificities and may be used in the settings where AFP values cannot be obtained. The predictive models developed in the current study were unique from the previously published clinical risk scores. The present models focused on predicting the risk for developing HCC in a short time window (6 months from each follow-up visit), while previous risk scores focused on providing an overall risk over a relatively long time period, such as 3 years [7, 8, 11, 12], 5 years [7,8,9,10,11,12], 8 years [11], or 10 years [9, 10]. Given the difference in prediction time points, comparison between the performance of the present models and previous predictive models was not feasible. Because the present models predict the short-term risk, they have potential clinical implication in adjusting follow-up intervals and HCC surveillance intervals for individual patients. According to the current guidelines, the recommendation of a 6-month surveillance interval is primarily based on the cost-effectiveness of studies in the entire CHB patient population [15]. Our models may be used to tailor surveillance strategy by identifying patients at a high risk for HCC, even before any focal liver observation is detected by ultrasound. On the contrary, if the predicted risk of developing HCC in the next 6 months is very low, deferring the surveillance program may be considered in order to reduce the cost of investigation during follow-up visit.

Performance evaluation of the models on both the derivation dataset and external validation dataset is considered a strength of our study. When tested on an independent cohort in other different hospitals, the models performed consistently well, suggesting generalizability of the models. Of note, while our derivation dataset was collected systematically by the retrospective cohort method, the external validation dataset was collected by the case–control method because there was no systematic way to obtain a large dataset of patient cohort from the hospital where the external validation dataset was collected. This difference in data collection methods between the derivation and external validation datasets may explain the difference in proportions of patients who developed HCC (4.9% in the derivation dataset and 35.2% in the external validation dataset). Generally, cohort data are better than case–control data at validating performance of clinical predictive models. Nonetheless, collecting the external validation data by a case–control method did have an advantage as we were able to manually review and verify the correctness of all laboratory and clinical parameters within each record.

Surprisingly, the model incorporating AFP as a feature achieved lower sensitivity in the external validation dataset compared with the derivative dataset. Further analysis of the datasets revealed a contrasting trend in median AFP levels of HCC patients between the two datasets (data not shown). In the derivative dataset, the median AFP level increased from 2.18 ng/mL at 6 months before the index follow-up visit, to 4.90 ng/mL at the index follow-up visit, and finally 18.17 ng/mL at the date of HCC diagnosis. Conversely, in the external validation dataset, the median AFP level of HCC patients remained relatively constant, with values of 3.78 ng/mL, 3.69 ng/mL, and 3.42 ng/mL at the respective time points. This observed discrepancy in AFP level trends may reflect a difference in the tumor biology of HCC between the two datasets, potentially leading to an unexpectedly reduced diagnostic yield in the external validation dataset when utilizing a model incorporating AFP as a feature.

There are some limitations to our study. First, our models did not include HBV treatments and compliance to treatment as input features; however, treatment success/failure and drug compliance of patients can be mostly reflected through other laboratory parameters, for example, HBV viral load. Second, the derivation dataset was collected from a single institution which would affect training data variability. Collecting training data from more institutions would further improve the robustness and generalizability of the models. Although HCV status presented a low value of Pearson correlation coefficient (0.024) and was not in a set of selected features, we reviewed medical records of all included patients with anti-HCV positivity in order to confirm this unexpected result. Of the 65 patients with anti-HCV positivity in the derivation dataset, there were 10 patients (15.3%) diagnosed with HCC later. At the time of HCC diagnosis, 5 (50%) and 3 (30%) of those were in undetectable viral status and sustained virological response (SVR) status after treatment while the other 2 (20%) patients had active infection due to treatment refusal. Among 55 (84.7%) non-HCC patients in this group, the number of patients in undetectable viral status and in SVR status after treatment were 34 (61.8%) and 19 (34.5%), respectively. Regarding the 2 (3.7%) remaining patients, one patient could not receive SVR status after treatment while another lost from follow-up. Therefore, we summarized that almost all cases with anti-HCV positivity in the derivative dataset did not have active HCV infection, and HCV status might not be a confounding factor for developing our models. Third, the external validation dataset was collected by the case–control method, which might not reflect the true incidence of HCC in the population. An incidence of HCC in the external validation dataset was higher than those in the general population of CHB-infected patients, that is, 35.2% vs. 0.3–3.7% [3]. This might affect the estimated performance of the models. Evaluating the model performance in a multicenter cohort with an average incidence of HCC is needed to ensure more accurate predictions. Lastly, our models utilized information from each single index follow-up to predict development of HCC, but did not utilize the changes in laboratory and clinical parameters over time. We believed that the information from a recent visit should be sufficient to predict the risk for HCC development in the short time window of 6 months. However, further prospective studies investigating the use of time-sequential models are of interest.

## Conclusions

Extreme gradient boosted models performed well in predicting risk of HCC development within 6 months. The models may be used to inform the short-term risk of HCC and tailor surveillance strategies in patients with CHB infection.
